# Dicyclo­hexyl­ammonium bromide

**DOI:** 10.1107/S1600536808023155

**Published:** 2008-07-31

**Authors:** Kong Mun Lo, Seik Weng Ng

**Affiliations:** a Department of Chemistry, University of Malaya, 50603 Kuala Lumpur, Malaysia

## Abstract

In the title compound, C_12_H_24_N^+^·Br^−^, both cyclo­hexane rings adopt the usual chair conformation. The cation and anion are linked by N—H⋯Br hydrogen bonds into a linear chain running along the *c* axis.

## Related literature

For the crystal structure of dicyclo­hexyl­ammonium chloride, which belongs to the space group *P*2_1_/*c*, see: Ng (1995 ▶).
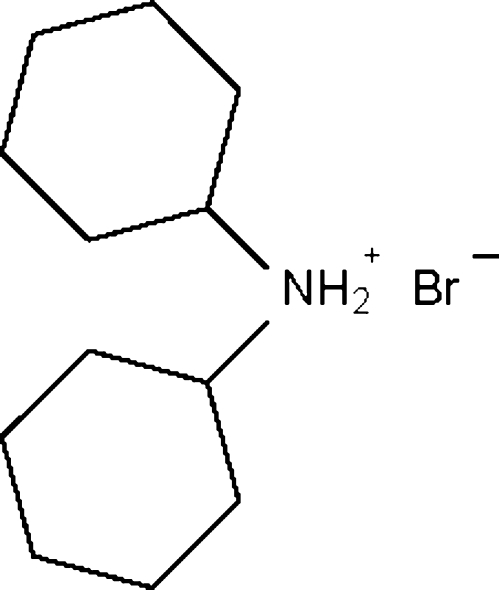


## Experimental

### 

#### Crystal data

C_12_H_24_N^+^·Br^−^
*M* *_r_* = 262.23
Orthorhombic, 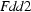

*a* = 24.1258 (4) Å
*b* = 39.3926 (7) Å
*c* = 5.4878 (1) Å
*V* = 5215.49 (16) Å^3^
*Z* = 16
Mo *K*α radiation
μ = 3.12 mm^−1^
*T* = 100 (2) K
0.40 × 0.02 × 0.02 mm

#### Data collection

Bruker SMART APEXII diffractometer
Absorption correction: multi-scan (*SADABS*; Sheldrick, 1996 ▶) *T*_min_ = 0.368, *T*_max_ = 0.940
15058 measured reflections
2961 independent reflections
2483 reflections with *I* > 2σ(*I*)
*R*_int_ = 0.049

#### Refinement

*R*[*F* ^2^ > 2σ(*F* ^2^)] = 0.035
*wR*(*F* ^2^) = 0.139
*S* = 0.98
2961 reflections
127 parameters
1 restraint
H-atom parameters constrained
Δρ_max_ = 0.67 e Å^−3^
Δρ_min_ = −0.56 e Å^−3^
Absolute structure: Flack (1983 ▶), with 1311 Friedel pairs
Flack parameter: 0.01 (2)

### 

Data collection: *APEX2* (Bruker, 2007 ▶); cell refinement: *SAINT* (Bruker, 2007 ▶); data reduction: *SAINT*; program(s) used to solve structure: *SHELXS97* (Sheldrick, 2008 ▶); program(s) used to refine structure: *SHELXL97* (Sheldrick, 2008 ▶); molecular graphics: *X-SEED* (Barbour, 2001 ▶); software used to prepare material for publication: *publCIF* (Westrip, 2008 ▶).

## Supplementary Material

Crystal structure: contains datablocks global, I. DOI: 10.1107/S1600536808023155/ci2641sup1.cif

Structure factors: contains datablocks I. DOI: 10.1107/S1600536808023155/ci2641Isup2.hkl

Additional supplementary materials:  crystallographic information; 3D view; checkCIF report

## Figures and Tables

**Table 1:** Hydrogen-bond geometry (Å, °)

*D*—H⋯*A*	*D*—H	H⋯*A*	*D*⋯*A*	*D*—H⋯*A*
N1—H11⋯Br1^i^	0.88	2.43	3.305 (5)	177
N1—H12⋯Br1	0.88	2.43	3.310 (5)	176

Symmetry code: (i) 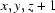.

**Fig. 1.**
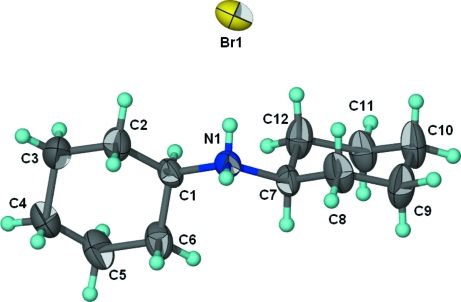
Displacement ellipsoid plot (Barbour, 2001) of [(C6H11)2NH2]+.Br- at the 70% probability level. Hydrogen atoms are drawn as spheres of arbitrary radius.

## supplementary crystallographic information

### Comment

In the title compound (Fig.1) both cyclohexane rings adopt the usual chair conformation. In the crystal structure, the cation and anion are linked by N—H···Br hydrogen bonds (Table 1) into a linear chain running along the *c* axis.

### Experimental

This compound was obtained as a side product from the reaction between dicyclohexylammonium bis(chlorodifluroacetato)cyclopentyldiphenystannate (0.5 g, 0.6 mmol) and 4-dimethylaminopyridine hydrobromide perbromide (0.23 g, 0.6 mmol) in a mixture of chloroform and ethanol. Crystals were obtained upon evaporation of the solvent.

### Refinement

H atoms were placed in calculated positions (N—H = 0.88 Å and C—H = 0.99–1.00 Å) and were included in the refinement in the riding model approximation, with U_iso_(H) = 1.2*U*_eq_(C,N).

### Crystal data

**Table:**

C_12_H_24_N^+^·Br^–^	*F*_000_ = 2208
*M**_r_* = 262.23	*D*_x_ = 1.336 Mg m^−^^3^
Orthorhombic, *F**d**d*2	Mo *K*α radiation λ = 0.71073 Å
Hall symbol: F 2 -2d	Cell parameters from 3733 reflections
*a* = 24.1258 (4) Å	θ = 2.7–23.5º
*b* = 39.3926 (7) Å	µ = 3.12 mm^−^^1^
*c* = 5.4878 (1) Å	*T* = 100 (2) K
*V* = 5215.49 (16) Å^3^	Prism, colourless
*Z* = 16	0.40 × 0.02 × 0.02 mm

### Data collection

**Table:**

Bruker SMART APEXII diffractometer	2961 independent reflections
Radiation source: fine-focus sealed tube	2483 reflections with *I* > 2σ(*I*)
Monochromator: graphite	*R*_int_ = 0.049
*T* = 100(2) K	θ_max_ = 27.5º
ω scans	θ_min_ = 2.0º
Absorption correction: multi-scan(SADABS; Sheldrick, 1996)	*h* = −31→31
*T*_min_ = 0.368, *T*_max_ = 0.940	*k* = −50→50
15058 measured reflections	*l* = −7→6

### Refinement

**Table:**

Refinement on *F*^2^	Hydrogen site location: inferred from neighbouring sites
Least-squares matrix: full	H-atom parameters constrained
*R*[*F*^2^ > 2σ(*F*^2^)] = 0.035	*w* = 1/[σ^2^(*F*_o_^2^) + (0.1*P*)^2^ + 1*P*] where *P* = (*F*_o_^2^ + 2*F*_c_^2^)/3
*wR*(*F*^2^) = 0.139	(Δ/σ)_max_ = 0.001
*S* = 0.98	Δρ_max_ = 0.67 e Å^−^^3^
2961 reflections	Δρ_min_ = −0.56 e Å^−^^3^
127 parameters	Extinction correction: none
1 restraint	Absolute structure: Flack (1983), with 1311 Friedel pairs
Primary atom site location: structure-invariant direct methods	Flack parameter: 0.01 (2)
Secondary atom site location: difference Fourier map	

### Fractional atomic coordinates and isotropic or equivalent isotropic displacement parameters (Å^2^)

**Table:**

	*x*	*y*	*z*	*U*_iso_*/*U*_eq_	
Br1	0.057495 (18)	0.153140 (12)	0.50000 (6)	0.03906 (17)	
N1	−0.01800 (15)	0.14541 (8)	1.0006 (10)	0.0288 (7)	
H11	0.0030	0.1479	1.1305	0.035*	
H12	0.0036	0.1475	0.8724	0.035*	
C1	−0.05883 (19)	0.17402 (9)	0.9961 (13)	0.0304 (8)	
H1	−0.0854	0.1705	0.8584	0.037*	
C2	−0.0271 (3)	0.20707 (12)	0.9555 (13)	0.0493 (16)	
H2A	−0.0068	0.2059	0.7988	0.059*	
H2B	0.0004	0.2102	1.0875	0.059*	
C3	−0.0668 (4)	0.23723 (14)	0.9517 (13)	0.060 (2)	
H3A	−0.0454	0.2585	0.9340	0.072*	
H3B	−0.0919	0.2352	0.8098	0.072*	
C4	−0.1006 (3)	0.23867 (14)	1.1826 (12)	0.0534 (17)	
H4A	−0.0758	0.2438	1.3216	0.064*	
H4B	−0.1280	0.2573	1.1697	0.064*	
C5	−0.1307 (2)	0.20596 (13)	1.2315 (17)	0.0496 (14)	
H5A	−0.1592	0.2024	1.1044	0.060*	
H5B	−0.1498	0.2075	1.3909	0.060*	
C6	−0.0908 (2)	0.17545 (11)	1.2338 (15)	0.0428 (12)	
H6A	−0.0646	0.1776	1.3718	0.051*	
H6B	−0.1121	0.1542	1.2557	0.051*	
C7	−0.04080 (18)	0.10966 (10)	1.0028 (13)	0.0317 (9)	
H7	−0.0664	0.1068	1.1448	0.038*	
C8	0.0077 (2)	0.08534 (12)	1.0283 (12)	0.0442 (13)	
H8A	0.0272	0.0898	1.1839	0.053*	
H8B	0.0343	0.0892	0.8937	0.053*	
C9	−0.0117 (3)	0.04860 (12)	1.0227 (15)	0.0514 (15)	
H9A	−0.0363	0.0442	1.1637	0.062*	
H9B	0.0207	0.0333	1.0359	0.062*	
C10	−0.0429 (3)	0.04108 (12)	0.7870 (14)	0.0489 (16)	
H10A	−0.0173	0.0432	0.6469	0.059*	
H10B	−0.0570	0.0175	0.7907	0.059*	
C11	−0.0911 (2)	0.06546 (12)	0.7555 (15)	0.0438 (12)	
H11A	−0.1185	0.0614	0.8864	0.053*	
H11B	−0.1094	0.0609	0.5976	0.053*	
C12	−0.0729 (2)	0.10264 (11)	0.7628 (15)	0.0404 (11)	
H12A	−0.1058	0.1176	0.7536	0.048*	
H12B	−0.0488	0.1076	0.6212	0.048*	

### Atomic displacement parameters (Å^2^)

**Table:**

	*U*^11^	*U*^22^	*U*^33^	*U*^12^	*U*^13^	*U*^23^
Br1	0.0308 (2)	0.0537 (3)	0.0327 (2)	−0.0001 (2)	0.0004 (2)	−0.0078 (2)
N1	0.0362 (18)	0.0267 (16)	0.0236 (17)	0.0004 (13)	−0.003 (2)	−0.003 (2)
C1	0.038 (2)	0.0261 (17)	0.0277 (19)	0.0028 (17)	−0.003 (2)	−0.007 (3)
C2	0.062 (3)	0.028 (2)	0.058 (4)	−0.002 (2)	0.023 (3)	−0.002 (2)
C3	0.104 (5)	0.031 (2)	0.044 (4)	0.008 (3)	0.015 (4)	0.002 (2)
C4	0.067 (4)	0.038 (3)	0.056 (4)	0.010 (3)	0.015 (3)	−0.010 (3)
C5	0.042 (3)	0.042 (3)	0.065 (4)	0.007 (2)	0.004 (4)	−0.013 (3)
C6	0.032 (2)	0.030 (2)	0.066 (4)	0.0003 (17)	0.014 (3)	−0.006 (3)
C7	0.037 (2)	0.0227 (17)	0.036 (2)	−0.0002 (15)	0.000 (3)	−0.005 (2)
C8	0.052 (3)	0.039 (2)	0.042 (3)	0.012 (2)	−0.023 (3)	−0.013 (3)
C9	0.070 (4)	0.029 (2)	0.055 (4)	0.010 (2)	−0.025 (4)	0.000 (3)
C10	0.055 (3)	0.026 (2)	0.066 (5)	0.005 (2)	−0.020 (3)	−0.013 (3)
C11	0.046 (3)	0.032 (2)	0.053 (3)	0.0006 (19)	−0.014 (4)	−0.013 (3)
C12	0.042 (2)	0.027 (2)	0.052 (3)	−0.0005 (18)	−0.018 (3)	−0.002 (3)

### Geometric parameters (Å, °)

**Table:**

N1—C1	1.497 (5)	C6—H6A	0.99
N1—C7	1.512 (5)	C6—H6B	0.99
N1—H11	0.88	C7—C8	1.520 (7)
N1—H12	0.88	C7—C12	1.552 (9)
C1—C6	1.517 (10)	C7—H7	1.00
C1—C2	1.527 (6)	C8—C9	1.522 (7)
C1—H1	1.00	C8—H8A	0.99
C2—C3	1.527 (8)	C8—H8B	0.99
C2—H2A	0.99	C9—C10	1.525 (10)
C2—H2B	0.99	C9—H9A	0.99
C3—C4	1.508 (9)	C9—H9B	0.99
C3—H3A	0.99	C10—C11	1.517 (7)
C3—H3B	0.99	C10—H10A	0.99
C4—C5	1.503 (9)	C10—H10B	0.99
C4—H4A	0.99	C11—C12	1.529 (6)
C4—H4B	0.99	C11—H11A	0.99
C5—C6	1.540 (6)	C11—H11B	0.99
C5—H5A	0.99	C12—H12A	0.99
C5—H5B	0.99	C12—H12B	0.99
			
C1—N1—C7	117.5 (3)	C1—C6—H6B	109.7
C1—N1—H11	107.9	C5—C6—H6B	109.7
C7—N1—H11	107.9	H6A—C6—H6B	108.2
C1—N1—H12	107.9	N1—C7—C8	107.9 (4)
C7—N1—H12	107.9	N1—C7—C12	109.9 (5)
H11—N1—H12	107.2	C8—C7—C12	110.5 (4)
N1—C1—C6	110.4 (5)	N1—C7—H7	109.5
N1—C1—C2	108.3 (4)	C8—C7—H7	109.5
C6—C1—C2	110.4 (4)	C12—C7—H7	109.5
N1—C1—H1	109.2	C7—C8—C9	111.1 (4)
C6—C1—H1	109.2	C7—C8—H8A	109.4
C2—C1—H1	109.2	C9—C8—H8A	109.4
C1—C2—C3	110.5 (5)	C7—C8—H8B	109.4
C1—C2—H2A	109.5	C9—C8—H8B	109.4
C3—C2—H2A	109.5	H8A—C8—H8B	108.0
C1—C2—H2B	109.5	C8—C9—C10	110.7 (5)
C3—C2—H2B	109.5	C8—C9—H9A	109.5
H2A—C2—H2B	108.1	C10—C9—H9A	109.5
C4—C3—C2	111.0 (5)	C8—C9—H9B	109.5
C4—C3—H3A	109.4	C10—C9—H9B	109.5
C2—C3—H3A	109.4	H9A—C9—H9B	108.1
C4—C3—H3B	109.4	C11—C10—C9	110.6 (5)
C2—C3—H3B	109.4	C11—C10—H10A	109.5
H3A—C3—H3B	108.0	C9—C10—H10A	109.5
C5—C4—C3	112.3 (5)	C11—C10—H10B	109.5
C5—C4—H4A	109.1	C9—C10—H10B	109.5
C3—C4—H4A	109.1	H10A—C10—H10B	108.1
C5—C4—H4B	109.1	C10—C11—C12	112.6 (4)
C3—C4—H4B	109.1	C10—C11—H11A	109.1
H4A—C4—H4B	107.9	C12—C11—H11A	109.1
C4—C5—C6	111.6 (5)	C10—C11—H11B	109.1
C4—C5—H5A	109.3	C12—C11—H11B	109.1
C6—C5—H5A	109.3	H11A—C11—H11B	107.8
C4—C5—H5B	109.3	C11—C12—C7	109.6 (5)
C6—C5—H5B	109.3	C11—C12—H12A	109.7
H5A—C5—H5B	108.0	C7—C12—H12A	109.7
C1—C6—C5	109.9 (6)	C11—C12—H12B	109.7
C1—C6—H6A	109.7	C7—C12—H12B	109.7
C5—C6—H6A	109.7	H12A—C12—H12B	108.2
			
C7—N1—C1—C6	67.5 (6)	C1—N1—C7—C8	−175.4 (6)
C7—N1—C1—C2	−171.5 (6)	C1—N1—C7—C12	64.0 (6)
N1—C1—C2—C3	−179.5 (5)	N1—C7—C8—C9	−177.9 (6)
C6—C1—C2—C3	−58.5 (7)	C12—C7—C8—C9	−57.7 (7)
C1—C2—C3—C4	56.2 (8)	C7—C8—C9—C10	57.9 (8)
C2—C3—C4—C5	−54.5 (8)	C8—C9—C10—C11	−56.1 (8)
C3—C4—C5—C6	54.4 (9)	C9—C10—C11—C12	55.9 (9)
N1—C1—C6—C5	177.4 (4)	C10—C11—C12—C7	−55.3 (8)
C2—C1—C6—C5	57.6 (6)	N1—C7—C12—C11	174.6 (4)
C4—C5—C6—C1	−55.7 (8)	C8—C7—C12—C11	55.6 (7)

### Hydrogen-bond geometry (Å, °)

**Table:**

*D*—H···*A*	*D*—H	H···*A*	*D*···*A*	*D*—H···*A*
N1—H11···Br1^i^	0.88	2.43	3.305 (5)	177
N1—H12···Br1	0.88	2.43	3.310 (5)	176

Symmetry codes: (i) *x*, *y*, *z*+1.

## References

[bb1] Barbour, L. J. (2001). *J. Supramol. Chem.* **1**, 189–191.

[bb2] Bruker (2007). *APEX2* and *SAINT* Bruker AXS Inc., Madison, Wisconsin, USA.

[bb3] Flack, H. D. (1983). *Acta Cryst.* A**39**, 876–881.

[bb4] Ng, S. W. (1995). *Acta Cryst.* C**51**, 2149–2150.

[bb5] Sheldrick, G. M. (1996). *SADABS* University of Göttingen, Germany.

[bb6] Sheldrick, G. M. (2008). *Acta Cryst.* A**64**, 112–122. 10.1107/S0108767307043930 18156677

[bb7] Westrip, S. P. (2008). *publCIF* In preparation.

